# 

**Published:** 1905-12-23

**Authors:** 


**NOTICE TO CORRESPONDENTS.**

All MSS., Letters, Books for Review, and other matters intended for the Editor should be addressed The Editor, "The Hospital," The Hospital Buildings, 28 & 29 Southampton Street, Strand, W.C.

The Editor cannot undertake to return rejected MS., even when accompanied by stamped directed envelope.

**Notice to Advertisers and Subscribers.**

All Advertisements, Orders for Copies of The Hospital, and Business Communications should be addressed to **THE MANAGER (Not to the Editor),** The Hospital, 28 & 29 Southampton Street, Strand London, W.C.

The Cost of Subscription, post free, is at follows:—Per week, 3½d.; per quarter, 3s. 3d.; per half-year, 6s. 6d.; per annum, 13s. Foreign and Colonial:—Per annum, 19s. 6d., payable, in all cases, in advance.

**NOTICE.—Vol. XXXVIII. of "The Hospital" April to September 1905), in handsome cloth binding, will shortly be on sale at the office of this Journal, price 10s. 6d. Cases for binding the Numbers contained in this Volume 2s. Index to Vol. XXXVIII., 3½d., post free.**

**The Monthly Part for December is now ready. Price 1s., by post, 1s. 3d.**

### BOOKS RECEIVED.

C. W. Deacon and Co.

"The Ladder of Pain." By George H. R. Dabbs, M.D.

H. J. Glaisher.

"Electrolysis in the Treatment of Facial and other Blemishes." By J. D. P. McLatchie, M.B.

Mather and Crowther, Limited.

"Practical Advertising, 1905-06."

Baillière, Tindall, and Cox.

"Treatise on Orthopædic Surgery." By E. H. Bradford, M.D., and R. W. Lovett, M.D.

"Lectures on Clinical Psychiatry." By Dr. Emil Kraepelin.

"Physical Diagnosis." By R. C. Cabot, M.D.

P. S. King and Son.

"A History of English Philanthropy." By B. Kirkman Gray.

Longmans and Co.

"Harrogate Waters, Baths, and Climate." By Drs. Bain and Edgecombe.

"Responsibility: An Address to Girls." By the Rev. E. E. Holmes.

T. Nelson and Sons.

"The Old Curiosity Shop."

"Uncle Tom's Cabin."

Swan, Sonnenschein and Co.

"Sanatoria for Consumptives." By F. R. Walters, M.D., M.R.C.P.

25 cases of pneumonia with 2 deaths; 25 cases of diphtheria with 3 deaths. Among many other operations appendicectomy was performed 21 times without a death. There were 29 amputations (12 being of the leg and 4 of the thigh) without a death. Of 12 cases of ovariotomy all were successful. The total number of operations was 886, and the number of deaths subsequent to operation was 27. We were unable to find an anæsthetic table.

### MEDICAL APPOINTMENTS AND VACANCIES.

### APPOINTMENTS.

J. BLAND-SUTTON, F.R.C.S.Eng., Surgeon to the Middlesex Hospital.

HAROLD BURROWS, M.B., F.R.C.S., Assistant Surgeon to the Seamen's Hospital, Greenwich.

ROCK CARLING, M.B., F.R.C.S., Assistant Surgeon to the Seamen's Hospital, Greenwich.

C. COLEY CHOYCE, M.D.Edin., F.R.C.S., Medical Superintendent of the Seamen's Hospital, Greenwich.

J. MICHELL CLARKE, M.D.Cantab., Chairman of the Medical Faculty in University College, Bristol, and Professor of Medicine.

SIDNEY H. CLARKE, M.A., M.B., B.C.Cantab., Assistant Medical Officer to the Newport Borough Asylum, Caerleon, Mon.

L. A. CLUTTERBUCK, M.B., B.S.Durh., M.R.C.P.Lond., Honorary Physician to the St. Marylebone General Dispensary.

ARTHUR EDMUNDS, M.B., M.S., F.R.C.S.Eng., Surgeon in charge of Out-patients at the Great Northern Central Hospital.

EDWARD FAWCETT, M.B., M.S.Edin., Dean of the Faculty of Medicine at University College, Bristol.

W. SAMPSON HANDLEY, M.S., M.D.Lond., F.R.C.S.Eng., Assistant Surgeon to the Middlesex Hospital.

CHARLES JAMES HEATON, M.R.C.S., L.R.C.P.Lond., Honorary Visiting Surgeon to the Royal Sea Bathing Hospital, Margate.

JOHN HOWSON RAY, Ch.M.Vict., F.R.C.S.Eng., Honorary Assistant Surgeon to the Manchester Royal Infirmary.

P. WATSON WILLIAMS, M.D.Lond., Lecturer on Diseases of the Nose and Throat at University College, Bristol.

WILLIAM WRIGHT, D.Sc., M.B., F.R.C.S., has been appointed Lecturer on Anatomy at the Middlesex Hospital Medical School.

### VACANCIES.

BRISTOL ROYAL INFIRMARY.—Pathologist, Bacteriologist, and Director of Clinical Laboratories. Also Medical Registrar and Surgical Registrar.

BRISTOL, UNIVERSITY COLLEGE, FACULTY OF MEDICINE.—Professor of Pathology.

CAMBRIDGE, BOROUGH OF.—Medical Officer of Health.

CARDIFF INFIRMARY.—Resident Medical Officer.

CHELSEA HOSPITAL FOR WOMEN, Fulham Road, S.W.—Resident Medical Officer.

EASTERN DISPENSARY, Leman Street, Whitechapel. Resident Medical Officer.

EAST LONDON HOSPITAL FOR CHILDREN AND DISPENSARY FOR WOMEN, Shadwell, E.—House Physician.

GLASGOW EYE INFIRMARY.—Pathologist and Bacteriologist.

LONDON THROAT HOSPITAL, 204 Great Portland Street, W. Honorary Anæsthetist.

MARGATE, ROYAL SEA BATHING HOSPITAL.—Honorary Assistant Visiting Surgeon.

NEW HOSPITAL FOR WOMEN, Euston Road.—Clinical Assistants (female).

NORTHAMPTON GENERAL HOSPITAL.—House Physician. Also Honorary Assistant Surgeon.

NOTTINGHAM GENERAL DISPENSARY.—Assistant Resident Surgeon.

NOTTINGHAM GENERAL HOSPITAL.—Assistant House Surgeon.

ROYAL LONDON OPHTHALMIC HOSPITAL, City Road, E.C.—Refraction Assistants.

RYDE, ROYAL ISLE OF WIGHT COUNTY HOSPITAL.—Resident House Surgeon.

SEAMEN'S HOSPITAL SOCIETY, Greenwich.—Two Assistant Physicians and one Assistant Physician or Surgeon for Diseases of the Throat, Nose, and Ear.

TOTTENHAM HOSPITAL, London, N.—Honorary Surgical Registrar.

UNIVERSITY OF LONDON.—Professor of Protozoology.

WAKEFIELD, WEST RIDING ASYLUM.—Pathologist and Assistant Medical Officer.

### Medical Appointments.

### SEAMEN'S HOSPITAL SOCIETY.

THE Committee of Management invite applications for the following appointments on the Staff of the Seamen's Hospital Society, with duties at the DREADNOUGHT HOSPITAL, GREENWICH, to which is attached the London School of Clinical Medicine:—

TWO ASSISTANT PHYSICIANS.

ONE ASSISTANT PHYSICIAN or SURGEON for DISEASES of the THROAT, NOSE, and EARS.

### IMPORTANT NOTICE.

A Useful Xmas Present for Nurses.

THE HOSPITAL

**Calendar & Notebook**

FOR

**1906.**

A Useful Xmas Present for Nurses.

**This compact and useful little Notebook, which is of a convenient size for the pocket, is in appearance an exact reproduction of THE HOSPITAL Journal in miniature and contains much information of value to nurses, including: Hints for nurses; How to advertise; How to reply to an advertisement; Hints on how to accept a post; Measures; Apothecaries' and other weights, &c., &c.**

### To Nurses.

THE HOSPITAL Calendar and Notebook will be forwarded **Post Free** on receipt of **Three Halfpenny Stamps.** Nurses requiring additional Copies can obtain One Dozen, post free, for Six Penny Stamps.

**BINDING CASES,** in New Pegamoid Leather Cloth, gilt lettered, specially designed to take **THE HOSPITAL Calendar and Notebook,** with loop for hanging from Wallet or Chatelaine with One Copy of the Calendar, **3½d.** post free. These Cases are strongly made, and can be used from year to year, as they do not contain the year nor any lettering other than the title of the Calendar.

---

The Diary will shortly be ready, and it is advisable to make immediate application to avoid disappointment, as the edition is limited and no reprints will be issued when it is exhausted.

*Address*—The Manager, Calendar Dept., THE HOSPITAL,
28 & 29 Southampton Street, Strand, London, W.C.

